# 3D printed customised external cranial plate in a patient with syndrome of trephined: ‘a case report’

**DOI:** 10.1186/s41205-021-00123-7

**Published:** 2021-11-12

**Authors:** Mee H., Greasley S., Whiting G., Harkin C., Oliver G., Marsden D., Andrews R., Sireau S., Price RD., Anwar F., Timofeev IS., Hutchinson PJ., White PA., Helmy A.

**Affiliations:** 1 Department of Clinical Neurosciences, University of Cambridge & Cambridge University Hospitals NHS Foundation Trust, Cambridge, UK; 2 Clinical Engineering Innovation Team, Department of Clinical Engineering, Cambridge University Hospitals NHS Foundation Trust, Cambridge, UK; 3 3D visualisation and printing department, Media Studios, Cambridge University Hospitals NHS Foundation Trust, Cambridge, UK; 4 Department of Plastic and Reconstructive Surgery, Cambridge University Hospitals NHS Foundation Trust, Cambridge, UK

## Abstract

**Background:**

Syndrome of the trephined is a well-recognised phenomenon that occurs in patients following a craniectomy. It is associated with several symptoms, including headaches, motor impairments, cognitive disorders and reduced consciousness. Treatment for the syndrome usually involves replacing the skull defect.

**Case Study:**

A 71-year-old male underwent a left-sided craniectomy after being diagnosed with biopsy-confirmed invasive squamous cell carcinoma with associated skull erosion. Subsequently, he developed a severe case of syndrome of the trephined (SoT,) resulting in having to lie flat to prevent the motor component of the Glasgow Coma Score (GCS) falling from M5/6 (E3/4 Vt M5/6) to M1 (E3/4 Vt M1) on sitting to 30 degrees. Unfortunately, due to ongoing chest sepsis and physical frailty, he was unable to undergo a cranioplasty. Therefore, to aid in clinical stabilisation, the treating physicians and clinical engineering teams designed and manufactured a prosthesis on-site, allowing rapid patient treatment. The prosthesis led to the patient being able to sit up to 30 degrees without the motor component of the GCS falling from M6 to M1 (E4 VT M6).

**Conclusion:**

Clinical improvements were demonstrated with definitive neurological improvement after applying the external cranial plate in clinical outcome measures and radiographically. Furthermore, we have shown that rapid prototyping technology provides a flexible solution to synthesise bespoke medical prostheses with the correct expertise and regulatory framework.

## Introduction

Decompressive craniectomy is a well-recognised neurosurgical strategy for reducing intracranial pressure, most commonly in the setting of traumatic brain injury and stroke [1], but also for invasive malignancy and infection. Following craniectomy, there is the potential to reconstruct the bony defect with a range of materials to protect the underlying brain, improve cosmesis and mitigate against the Syndrome of the Trephined (SoT). This is termed cranioplasty, typically an elective procedure following the resolution of the original pathology. Various materials have been utilised for reconstruction, including autologous bone, hydroxyapatite, polyetheretherketone (PEEK), polymethylmethacrylate (PMMA), and titanium. A few case reports [2, 3] report on applying an external prosthesis, using plaster as the material of choice for the treatment of SoT in patients who could not undergo a cranioplasty, as it is easy to manipulate into shape the bedside and quick to set. In our institution, Cambridge University Hospitals NHS Foundation Trust, we have a process for creating implantable custom-made 3D printed titanium cranial plates. These are prescribed by the clinician, designed in-house based on individual patient CT scans, then laser sintered in titanium alloy by a third-party manufacturer. Both the Trust and the manufacturer deliver this within ISO 13,485 quality management systems, and the plates are classed as custom made medical devices compliant with the Medical Devices Directive (93/42/EEC). We utilised this experience in plate design by applying the same principles in manufacturing the external prosthesis but using Formlabs Dental SG resin rather than titanium. Potential advantages of 3D printing in this context are being accurate, detailed, plate fitting but accepting a slightly longer time frame from design to the application than previously described methods using plaster.

SoT, or ‘sunken flap syndrome’, is a poorly understood complication of a craniectomy, with an incidence of around 13 % [4] but is very likely to be underestimated, given our limited understanding of the condition and under-reporting. It develops slowly over days or weeks following the craniectomy. However, it can result in marked neurological dysfunction, which usually improves following cranial reconstruction—first described by Grant and Norcross in 1939 [5] as a syndrome comprising of severe headaches, dizziness with pain at the craniectomy site and altered cognitive state of mind. These clinical manifestations of the syndrome are variable, and in the most severe cases result in marked deterioration of neurological state, leading to coma, prolonged admission to intensive care, with increased risk of secondary complications such as aspiration pneumonia and in the longer-term, potential increases in disability and associated dependency. However, there are three overarching features [6] that help identify the syndrome:

- Neurological deficits occurring days, weeks, or months after the craniectomy.
- The occurrence of neurological deficits separate to those associated with the initial pathology.
- Clinical resolution after cranioplasty.

Recognition of this syndrome can be clinically challenging, especially in those patients with prolonged disorders of consciousness (PDOC), where it should be considered if a sunken flap is present. In those with likely SoT, expedited cranioplasty is considered to reverse the neurological deficit and aid neurorehabilitation.

This report describes the case of a 71-year-old patient who was suffering from severe sunken flap syndrome following a craniectomy and who was not suitable for an immediate cranioplasty due to chest sepsis and underlying physical frailty as a result of his malignancy and co-morbidities, with a very high risk of mortality associated with further anaesthesia and surgical intervention. However, he was managed by successfully manufacturing a novel external cranial plate device to improve his symptoms. To our knowledge, this is the first description of an external prosthesis manufactured using rapid prototyping technology to be used for an external cranioplasty, but it is a widely practised manufacturing process for internal cranioplasties.

## Case Study

### Initial presentation and plate indication

A 71-year-old male presented to a dermatology clinic in July 2019 with a left frontal scalp mass and was diagnosed with biopsy-confirmed, non-metastatic, invasive squamous cell carcinoma with associated erosion of the skull. In August 2019, after a comprehensive multi-disciplinary team discussion, the patient underwent an en bloc resection of the lesion, including large left-sided craniectomy and resection of the underlying involved dura. The defect was reconstructed with a subsequent right free flap latissimus dorsi muscle/serratus anterior fascial flap transfer. Post-operatively, the patient was managed in the neuro-intensive care unit (NCCU) before being transferred to the neurosurgical ward. Post-operatively the patient remained mildly confused, with a GCS of 14/15 but developed pneumonia and was re-admitted to the NCCU for respiratory support.

Over the following week, a sunken brain flap developed concordant with the drop in GCS. Laying the bed flat to mitigate the sunken flap resulted in an improvement in GCS while raising the head of the bed to 30 degrees resulted in a GCS motor score reduction of M6 to M1 at worst. The patient was repeatedly challenged with the head of the bed raises over two weeks to assess for possible resolution of SoT. Unfortunately, given the ongoing risk of infection associated with chest sepsis and overall physical frailty, the patient could not undergo a cranioplasty reconstruction. In addition, laying the patient flat was considered an additional barrier to respiratory management. Given the limited therapeutic options in this circumstance, following a discussion with Clinical Engineering, the hospital Medical Devices Advisory Group were approached to conduct an n=1 trial of external plate manufacture to mitigate the symptoms of the SoT as a bridge to definitive cranioplasty insertion. The clinical objective was to stabilise the patient’s condition, enabling the patient to sit up without neurological compromise and potentially expedite the removal of the tracheostomy and transfer out of the NCCU.

### Medical device development and application

Clinical Engineering Innovation (CEI) is a team of NHS-funded engineers based within the hospital who design and manufacture innovative medical technologies to solve unmet clinical needs for patient benefit. The aim was to design and manufacture a device to replicate the effects of a cranioplasty, thus reducing the neurological sequelae of SoT.

Following a multi-disciplinary discussion between CEI and the clinical team, a plan was made to design and manufacture a custom 3D printed external cranial plate, building on the in-house expertise within the relevant departments. A detailed prescription was obtained from the consultant, who also approved the 3D model of the plate before printing. Informed consent was obtained from the patient’s next of kin, and it was explained that this was a novel device of unknown benefit. The scientific justification for this solution was based on the known success of traditional implantable cranial plates[6], with a minimal number of previous case reports demonstrating similar applications [2]. The CEI team conducted a detailed risk assessment of the proposed external cranial plate with input from clinical colleagues. The likelihood and consequence of each hazard were scored in a risk evaluation table, and risk mitigations were identified to reduce risks to an acceptable level. The hazards considered were biological, mechanical and human factors. A biocompatible material was selected, which was formulated for dental applications. Silicone adhesive dressings were used to separate the plate from the patient’s skin to mitigate biocompatibility risks and protect the wound site. The plate thickness was specified to reduce the risk of plate shattering. The patient was closely monitored, and the protocol stated that the plate should be removed after a maximum of 72 h or earlier if clinically indicated. Manufacture & quality checking processes were also implemented to mitigate risks by inspecting sharp edges and stress propagation points. Based on this risk assessment, the multi-disciplinary team concluded that residual risks of a specifically designed external cranial plate were very low, and the intervention offered potential benefits to the patient.

**Fig. 1:**
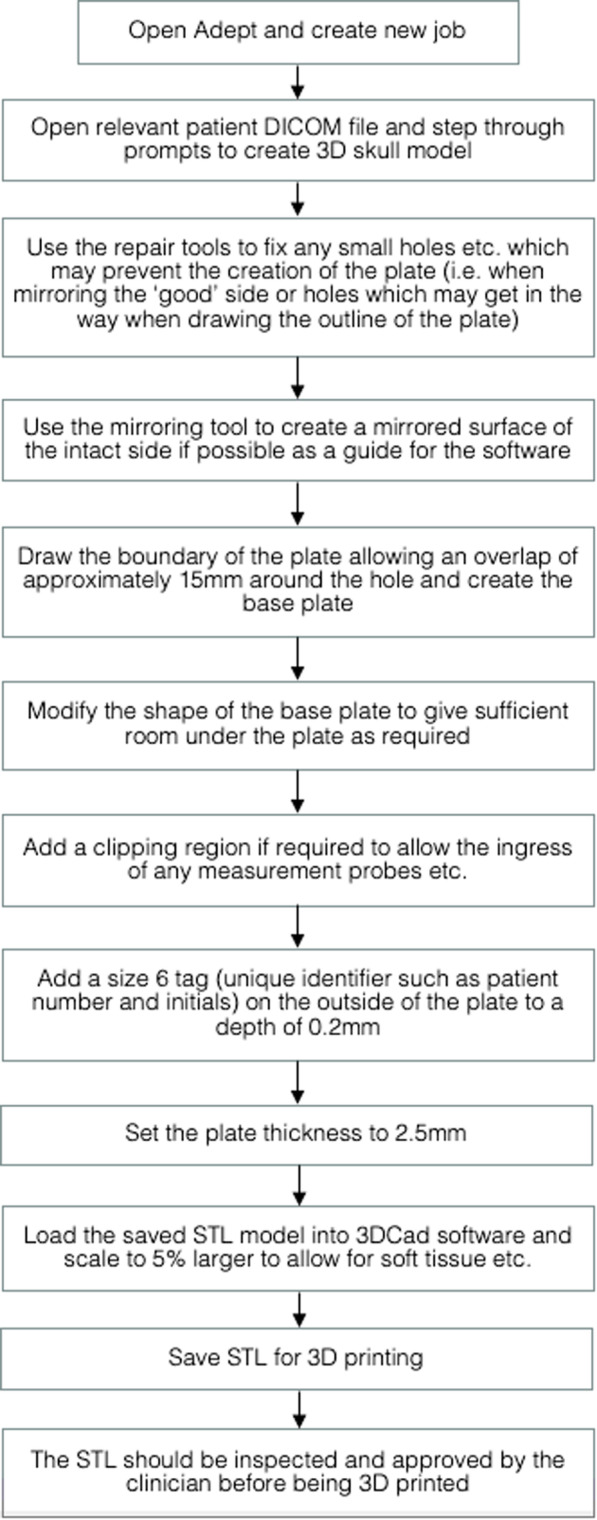
Workflow from CT image file to printable STL file.

ADEPT software is used routinely within the Trust to design custom implantable cranial plates from CT scans. This was used to design the external cranial plate following an adapted version of the Trust’s standard process for implantable cranial plates, as described in Fig. 1. A mirrored model of the intact side of the skull formed the basis of the plate. In addition, the design was modified to allow the free-graft pedicle to pass beneath the plate without compression, and the 3D model was enlarged to allow space for silicon adhesive dressings to be applied circumferentially on the patient’s skin to separate the plate from the patient’s skin and any wounds at the surgical site. The prescribing clinician approved the 3D model of the final plate design before being sent to print.

**Fig. 2:**
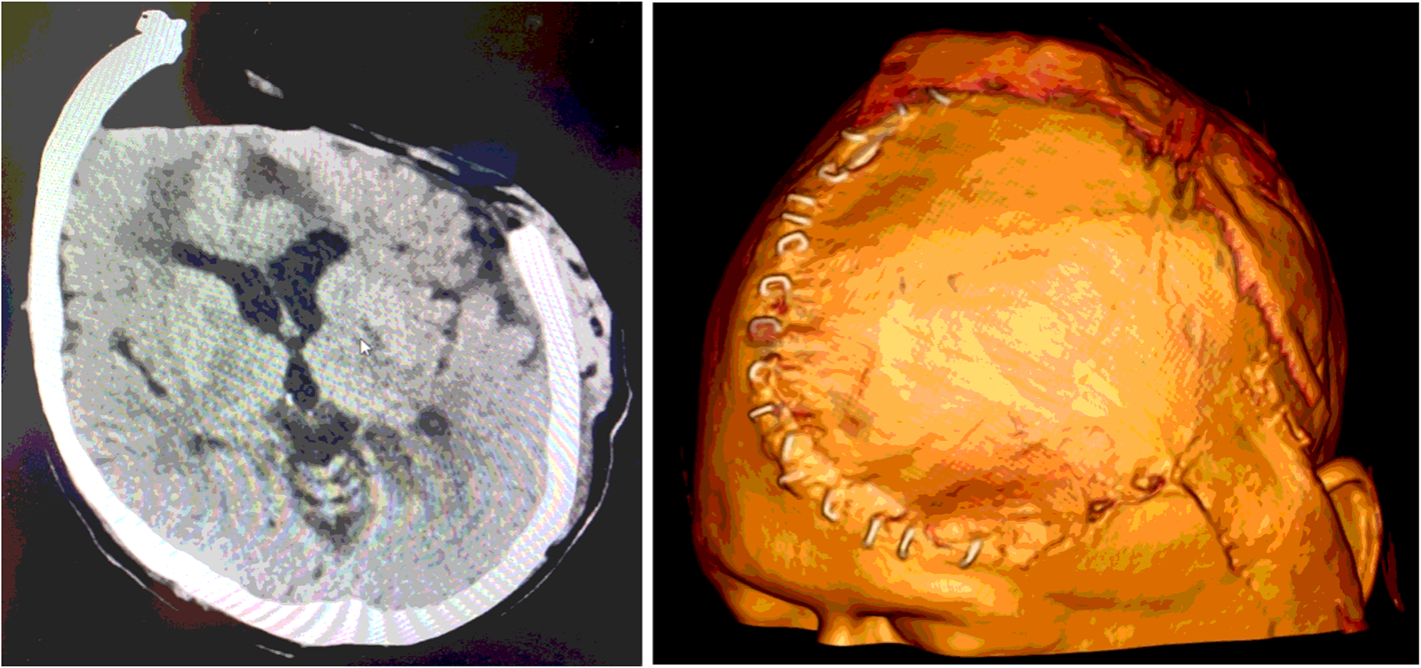
CT head scan with 3D reconstruction before device application

The plate was printed in biocompatible dental SG resin on a Formlabs Form 2 3D printer, cured and finished by the CEI team, then sterilised by the in-house sterile services department. Formlabs Dental SG resin was chosen because the material data sheet specifies that the material is biocompatible (adheres to the EN ISO 10993-1:2009/AC:2010 standard), and it is suitable for steam sterilisation in an autoclave. Formlabs “Instructions for Use for Dental SG” and “Application Guide for 3D Printing Surgical Guides with the Form 2” were used to guide all stages of the manufacturing and sterilisation process. PreForm software was used to align and orientate the plate model to minimise warping, prevent the occurrence of minima and cupping, generate the support structure attached to the outside surface of the plate, and reduce the thickness of the support touchpoints to minimise surface blemishing. The plate was printed using a resin tank and a platform dedicated to prints in Dental SG resin. After printing, the plate was removed from the printer, rinsed twice in fresh 90 % isopropyl alcohol for 10 min, then air-dried before curing for 30-40 min in a UV curing oven. The support structure was removed using flush cutters, and then the plate was inspected for stress propagation points and sharp edges. The outer surface was finished with fine sandpaper to remove any support remnants, then the plate was inspected and accepted by a member of the CEI team and sent to the in-house sterilisation department. The plate was steam-sterilised in an autoclave at 134 °C per local protocols, then packaged and labelled ready for use. As described in the Dental SG resin instructions for use, material colour changes indicated that each step was completed to create a sterile, biocompatible finished product (Fig. 2).

The entire design and manufacture process was overseen by a multi-disciplinary team consisting of clinicians and engineers. CEI was responsible for all necessary technical documentation for a custom-made medical device for in-house manufacture and used the Medical Device Regulation 2017/475, working within a quality system accredited to ISO 9001 and ISO 13,485. This documentation included the prescription from the clinician, patient consent form, risk assessment, technical drawings of the custom device, Formlabs Dental SG material data sheet, custom external cranial plate instructions for use, sterilisation documentation, device labels, checklist demonstrating conformance of the device to the general safety and performance requirements set out in Annex I of the Medical Device Regulation 2017/475, and a statement of conformity (as described in Annex XIII of the Medical Device Regulation 2017/475),). The collaboration between the clinicians and the in-house CEI team, combined with the Trust’s existing processes for implantable titanium cranial plates, enabled the whole process from inception to application of the device to occur in less than one week, which would not have been the case if the clinicians had worked with an external contractor rather than the internal CEI team.

Indications for plate removal, associated with possible harm, were agreed with the Medical Devices Advisory Group, including lack of improvement after 72 h or further deterioration of the patient’s GCS. Therefore, a plan was made to incline the patient’s bed over 24 h gradually, and success would be measured by the recording of the GCS and a CT scan of the brain. In addition, a plan was made to remove the device after 72 h to replace wound dressings and check the viability of the skin. The observation was carried out in a high dependency environment with written advice to all relevant medical and nursing professionals.

**Fig. 3:**
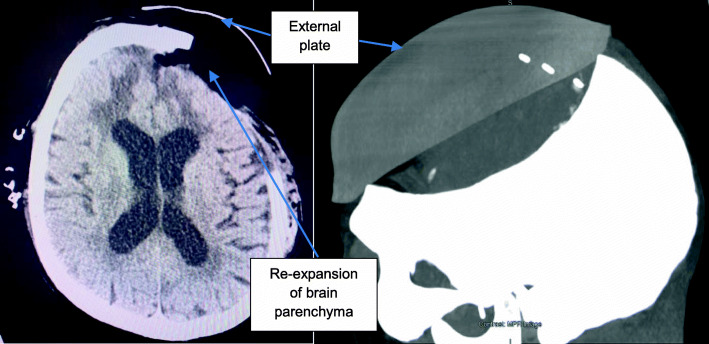
CT head scan with 3D reconstruction two days after device application. As the brain surface expands towards the inner surface of the cranioplasty, the ventricles become enlarged. This is an *ex vacuo* phenomenon and does not represent hydrocephalus

Adaptic Touch non-adhering silicone dressings were applied along the edges of the plate to act as a layer of protection between the plate and skin. Cosmopor E sterile absorbent adhesive dressings were then applied across the edges of the plate and the patient’s skin to secure the plate in place (Fig. 3).

### Clinical Response

Before the external plate application, the patient could not sit up to 30 degrees without a drop in the motor score on the GCS from M6 to M1. However, within 36 h of the device’s application, they could sit up to 45 degrees facilitating chest physio with no drop in the motor score. Initially, there were some difficulties with periods of hypotension, but these resolved and were thought to be related to the physiological effect of lying flat for a prolonged period before applying the external plate. The plate was removed for clinical reasons after three days, not related to the plate application as per the risk assessment plan.

**Fig. 4:**
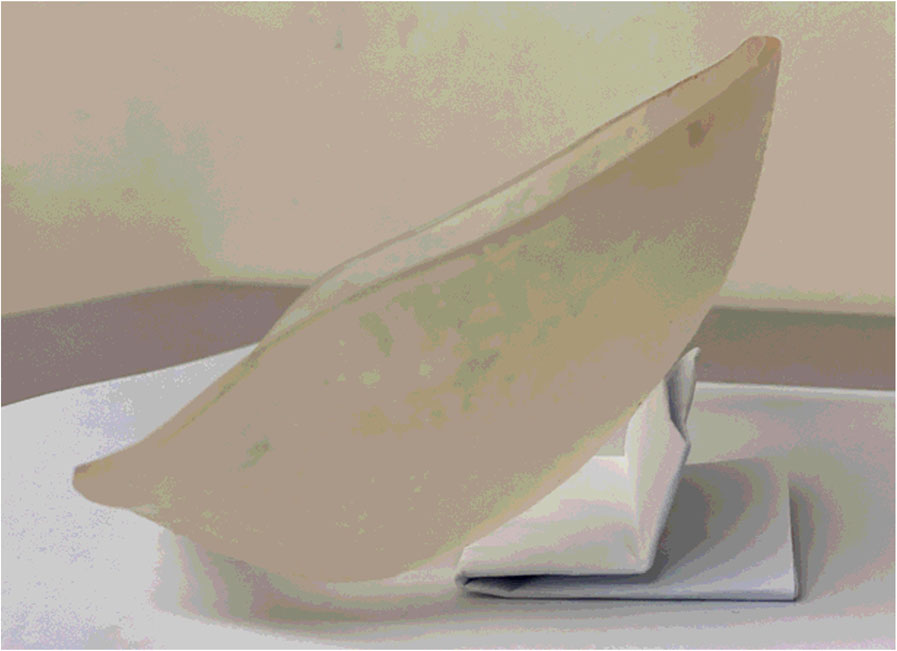
External cranioplasty before application

**Fig. 5:**
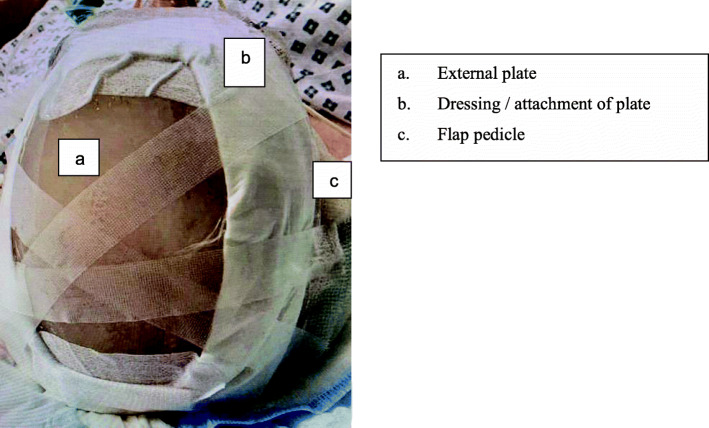
External plate after application

CT head imaging shows the intracranial changes, with the sunken flap before the device application (Fig. 4.) and brain tissue re-expansion (Fig. 5.) 2 days post device application.

Over the following week, the patient became increasingly drowsy again, dropping the motor score of the GCS from M6 to M5 while sitting at 45 degrees. Clinically, it was evident that the patient was again developing the symptoms of SoT but not to the same severity as the first occurrence. The plate was re-applied in the same fashion, and the opportunity was taken to carry out a more detailed neurological assessment utilising the Wessex Head Injury Matrix (WHIM).

The WHIM is an observational scale designed to monitor changes in an individual’s level of responsiveness and interactions as they regain consciousness. It is used primarily in patients who have suffered a traumatic brain injury (TBI) [7]. Although this patient had not suffered a TBI, it was decided that the WHIM would be an ideal tool to observe small but very relevant changes in neurology before and after the application of the device. Patients are scored against the hierarchal scale, and after ten non-observations in a row, the score of the last completed observation is used. Before re-application of the plate, the patient scored 22 on the WHIM. This was subsequently repeated 48 h after attachment of the plate, and the patient scored 38. As in the first application, the plate was removed after 72 h with the patient being clinically weak but neurologically stable sitting at 45 degrees.

The patient remained clinically stable over the following ten days, but increasingly frail secondary to his underlying pathology and subsequently developed a further chest infection. Despite active treatment, further clinical deterioration occurred, and it was decided to withdraw active care, and a palliative, end of life care approach was taken. The patient passed away peacefully a few days later.

## Discussion

This is the first report of an in house designed and manufactured 3D printed prosthesis used to help mitigate the symptoms of the Syndrome of the Trephined. In addition, it demonstrates how rapid prototyping technology can be flexibly applied to solve novel clinical problems within appropriate governance and regulatory framework.

Several proposed theories regarding the underlying pathophysiology of the syndrome of trephined are known, with likely multiple mechanisms involved. Firstly, altered external barometric pressures on the scalp result in increased pressure across the cerebral vasculature, leading to decreased cerebral blood flow and impaired CSF flow, resulting in neurological compromise [5]. This has been demonstrated using xenon-enhanced perfusion CT, in which, following craniectomy, cerebral blood flow (CBF) both on the ipsilateral and contralateral sides [8]. CBF, cerebral perfusion, and CSF circulation disturbances are well described in the chronic phase of acquired brain injury following a craniectomy. Worsening of these pathophysiological consequences due to the sunken flap is likely why a subset of patients develop the syndrome of trephined, and it is the cranioplasty that stabilises the atmospheric pressure gradient, re-establishing the fixed volume of the cranial vault allowing the brain parenchyma to re-expand with studies demonstrating improved CSF hydrodynamics [9–11] and improvements in CBF following a cranioplasty.

There was an unambiguous clinical improvement in GCS motor score following application of the cranial plate, which abated on the removal of the plate and improved once more on re-application. Although this is an n=1 study, and no definitive conclusions can be drawn, the reversible clinical response suggests that it was the application of the plate that affected the clinical improvement analogous to the insertion of conventional 'internal’ cranioplasty plates. We hypothesise that a combination of the plate and the dressings limited movement of the skin and subsequent sinking of the flap, reducing association distortion and recapitulating the normal CSF hydrodynamic pathways. Alternatively, sealing the defect from the effects of atmospheric pressure may have prevented cerebral blood flow and CSF flow impairment, minimising midline shift and brain slump and axonal stretch. Given that the definitive mechanism of SoT is not known, we recognise that these hypotheses are necessarily speculative.

Limitations include how best to secure the device to the craniectomy site; in this case, Adept dressings were used but in warm environments, and if the hair was present, this could prove difficult. Also, although the plate was manufactured within one week, this is still a significant period for a patient with neurological compromise, and other streamlined design and manufacturer processes would need to be considered in the future. Further definitive research is required to better understand the potential benefits of the external cranioplasty and its pathophysiological consequences concerning the syndrome of trephined.

## Conclusions

This study has two important implications. Firstly, while 3D printing has been proposed as a new method for the bespoke design of prostheses in various settings [8, 12], we believe unique and challenging cases such as this within the clinical arena can provide the most significant potential benefit. This novel, safe and inexpensive medical device was designed and made promptly to benefit the patient’s neurological state directly. Secondly, we have provided the first description of 3D printed external cranioplasty to aid in the clinical stabilisation of a patient suffering from the syndrome of trephined. This provides a wholly novel therapeutic strategy in neurorehabilitation following a craniectomy.

## Data Availability

N/A.
